# The role of adrenaline in cardiopulmonary resuscitation

**DOI:** 10.1186/s13054-018-2058-1

**Published:** 2018-05-29

**Authors:** Christopher J. R. Gough, Jerry P. Nolan

**Affiliations:** 1 Anaesthesia and Intensive Care Medicine, Royal United Hospital, Bath, BA1 3NG UK; 2 Resuscitation Medicine, Bristol Medical School, University of Bristol, Bristol, UK

**Keywords:** Cardiac arrest, Cardiopulmonary resuscitation, Adrenaline, Epinephrine, Outcome

## Abstract

Adrenaline has been used in the treatment of cardiac arrest for many years. It increases the likelihood of return of spontaneous circulation (ROSC), but some studies have shown that it impairs cerebral microcirculatory flow. It is possible that better short-term survival comes at the cost of worse long-term outcomes. This narrative review summarises the rationale for using adrenaline, significant studies to date, and ongoing research.

## Background

Adrenaline has been included in resuscitation guidelines worldwide since the 1960s and, through its action of increasing coronary and cerebral perfusion pressure, is thought to increase the chance of restoring a heartbeat (return of spontaneous circulation (ROSC)) and of improving long-term neurological outcome. However, there are no human data to show that long-term neurological outcome is improved with injection of adrenaline during cardiac arrest. Several observational studies document an association between the injection of adrenaline and worse neurological outcome, but all of these are confounded because of indication bias (those with more prolonged cardiac arrests are more likely to be given adrenaline and are more likely to have a poor outcome). This narrative review summarises the rationale for using adrenaline, significant studies to date, and ongoing research.

## Why is adrenaline used in cardiac arrest and why might it be harmful?

Adrenaline has been a key component of advanced life support algorithms for many years. Its mechanism of action—stimulation of α_1_ receptors in vascular smooth muscle—causes vasoconstriction. This increases the aortic diastolic pressure, which increases coronary perfusion pressure (CPP) and cerebral perfusion pressure (CePP). The CPP is strongly associated with return of spontaneous circulation (ROSC) [1].

Although global cerebral and coronary blood flow is increased by adrenaline, microcirculatory flow may be reduced. Once ROSC has been achieved, excessive plasma concentrations of adrenaline will cause tachycardia (which increases oxygen demand) and arrhythmias, including ventricular tachycardia and ventricular fibrillation (VF).

## Animal studies

A study of 36 adult pigs, which were randomised to one of two adrenaline doses (20 or 30 μg/kg) or to placebo, bolused every 3 minutes, documented increased arterial blood pressure and increased CePP in the adrenaline groups [2]. These two groups, however, had lower SpO_2_ values and lower cerebral tissue oximetry values than the placebo group, consistent with reduced organ and brain perfusion. A six-pig study measuring cerebral, coronary, and aortic pressures and blood flow identified that injection of 40 μg/kg of intravenous (IV) adrenaline significantly increased mean aortic pressure (29 ± 5 vs 42 ± 12 mmHg, *p* = 0.01), cerebral perfusion pressure (12 ± 5 vs 22 ± 10 mmHg, *p* = 0.01) and coronary perfusion pressure (8 ± 7 vs 17 ± 4 mmHg, *p* = 0.02), but mean coronary blood flow decreased (29 ± 15 vs 14 ± 7.0 mL/min, *p* = 0.03) [3].

Microcirculatory blood flow was evaluated with orthogonal polarization spectral imaging in ten pigs that were randomised to receive either adrenaline 30 μg/kg or vasopressin 0.4 units/kg during CPR [4]. Post-resuscitation microvascular flows and cerebral oxygen tension (PbO_2_) were higher and cerebral carbon dioxide tension (PbCO_2_) lower after vasopressin compared with adrenaline. In another study by the same group, cerebral blood flow (CBF; assessed with microcirculatory imaging), cerebral oxygen tension (PbO_2_), and carbon dioxide tension (PbCO_2_) were measured in four groups of five pigs. The pigs receiving bolus adrenaline (30 μg/kg) achieved a higher mean aortic pressure than those given placebo during and after CPR (*p* < 0.05), but had lower PbO_2_ values (*p* < 0.01) and higher PbCO_2_ values (*p* < 0.01) after resuscitation [5]. Microcirculatory blood flow was lower in the adrenaline groups than the placebo group after resuscitation (*p* < 0.01). This was also observed in a separate study where 15 pigs were subjected to 5 min of VF, and 5 minutes of precordial compression before electrical defibrillation was attempted [6]. Microcirculatory blood flow was assessed in the sublingual mucosa at regular intervals, and CPP was also recorded. Six of the pigs received 1 mg of adrenaline after 1 min of precordial compression. Injection of adrenaline reduced microcirculatory blood flow (*p* < 0.05), which persisted for several minutes.

In another study, piglets were randomised to vasopressin, or vasopressin and adrenaline, with the adrenaline given by bolus (20 μg/kg) followed by infusion (10 μg/kg/min) [7]. Although the adrenaline with vasopressin group had higher mean blood pressure (*p* = 0.03) and CBF (*p* < 0.05) during CPR, after resuscitation the CBF was numerically 36% lower, although this was not statistically significant (*p* = 0.06). Neuronal injury and signs of disruption to the blood–brain barrier were both greater in the adrenaline group.

In a study of 20 adult dogs, coronary, cerebral and renal blood flow were measured, and cardiac tissue samples were taken for lactate concentration and myocardial adenosine 5′-triphosphate (ATP) [8]. The dogs were allocated randomly into two groups—those that received CPR alone and those that also received adrenaline (1 mg bolus then 0.2 mg/min). The adrenaline group had higher myocardial blood flow (48 ± 11 vs 21 ± 4 ml/min/100 g, *p* < 0.05), but lower renal blood flow (1 ± 0 vs 74 ± 23 ml/min/100 g, *p* < 0.01). There was no significant difference between the groups in ATP values but the adrenaline group had a higher lactate concentration in the epicardium (6.3 ± 0.6 vs 4.2 ± 0.6 nmol/mg, *p* < 0.05, with a rise from baseline of 5.6 ± 0.5 vs 3.8 ± 0.5 nmol/mg, *p* < 0.05). The higher lactate values associated with administration of adrenaline could reflect either increased myocardial oxygen demand and/or stimulation of glycolysis.

The effect on CBF of bolus adrenaline compared with an infusion was evaluated in 24 pigs that were randomised to receive either boluses of adrenaline every 3 min (20 μg/kg) or a bolus (20 μg/kg) followed by an infusion (10 μg/kg/min). CBF was monitored continuously. The adrenaline bolus groups had transient increases in CBF after each bolus, but the infusion group had higher CBF overall (*p* < 0.01) [9].

In summary, adrenaline increases the mean aortic pressure, but the effect on coronary and cerebral blood flow is inconsistent. In many cases, adrenaline reduces microcirculatory flow, even if global organ blood flow is either increased or unchanged. The different techniques used to monitor cerebral blood flow may contribute to differences in results.

## Human physiological studies

In an early study of 100 patients, to enable continuous pressure monitoring, during cardiac arrest invasive lines were placed into the right atrium via the subclavian vein and into the aortic arch via the femoral artery [1]. Twenty-four patients had ROSC. The maximal CPP was much higher in the patients who had ROSC, and no patient with a maximal CPP less than 15 mmHg had ROSC.

An observational study of regional cerebral oxygenation (rSO_2_) measured by near-infrared spectroscopy (NIRS) in 36 patients with in-hospital cardiac arrest documented rSO_2_ for 5 minutes before and after 89 doses of adrenaline [10]. Of note, 66.7% of patients received only a single dose of adrenaline. Excluding 33 adrenaline events that were preceded by a previous dose of adrenaline given in the 5-minute window, the effect on rSO_2_ of 56 doses was assessed. The mean rSO_2_ increased by 1.4% in the 5 minutes after adrenaline dosing compared to the 5 minutes before (95% confidence interval (CI) 0.41–2.40%, *p* = 0.006). However, the rSO_2_ values were already increasing by 0.88%/minute before injection of adrenaline and this trend was not significantly altered by the adrenaline (*p* = 0.583). Whether or not NIRS is sufficiently sensitive and reliable for detecting changes in regional cerebral oxygenation associated with adrenaline remains to be established [11].

Patients in cardiac arrest may transition from one rhythm to another, for example from PEA to VF, which may in turn give them a higher chance of achieving ROSC. In an Oslo study of 174 patients with out-of-hospital cardiac arrest (OHCA) with an initial rhythm of PEA, patients given adrenaline were significantly more likely to transition into a different rhythm (rate ratio = 1.6, *p* < 0.001) [12]. Although the rate of transition from PEA to ROSC increased markedly in the adrenaline group, the rate of transition from ROSC to VT/VF also increased (regression parameter = 0.3, *p* < 0.01), as well as from ROSC to PEA (regression parameter = 1.07, *p* < 0.01).

In summary, adrenaline increases CPP and this is associated with a higher rate of ROSC. However, adrenaline also increases instability and although it increases the likelihood of transition to ROSC, it also makes the patient more prone to develop arrhythmias, including VF.

## Propensity analysis

Several Japanese studies have documented associations between adrenaline use and short- and long-term outcomes. These observational studies are prone to considerable bias (e.g. patients successfully resuscitated early are much less likely to have received adrenaline) and a variety of statistical techniques are used to adjust for confounders. One such technique is propensity analysis; this is used when two groups of patients have dissimilar characteristics that could account for any observed difference in outcome. A score is calculated that is the probability that a patient would receive the treatment of interest, based on characteristics of the patient, treating clinician, and environment [13, 14]. Many observational studies of the management of OHCA, such as some of those from the Japanese nationwide OHCA registry, use propensity score matching, which creates two groups of study participants—one group that received the treatment of interest and the other that did not—while matching individuals with similar propensity scores. This approach has several limitations. Firstly, only the measured characteristics can be adjusted for, so any unmeasured confounders that affect treatment selection or outcome will not be corrected for. Secondly, the quality of the propensity model used will affect its outcome, as will the size and quality of the included data. Observational data cannot establish causal relationships or treatment effects, but appropriately used propensity analysis on a sufficient sample size can provide a useful approximation of the effect of an intervention.

## Clinical observational studies, including systematic reviews and meta-analyses

Among 417,188 OHCAs in the Japanese nationwide registry between 2005 and 2008, ROSC before hospital arrival was achieved in 18.5% of 15,030 patients who received adrenaline, and in 5.7% of 402,158 patients who did not receive adrenaline (*p* < 0.001; unadjusted odds ratio (OR) 3.75; 95% CI 3.59–3.91; Table 1) [15]. After propensity matching the adjusted odds ratio (aOR) for ROSC was 2.51 (95% CI 2.24–2.80). Although the raw outcome data indicate a higher rate of one-month survival in those receiving adrenaline, after propensity matching the aOR for one-month survival was 0.54 (95% CI 0.43–0.68) and for CPC 1–2 the aOR was 0.21 (95% CI 0.10–0.44). These data suggest that more patients who received adrenaline survived to hospital admission, but that longer-term outcomes were better in the no-adrenaline group.

**Table 1:** Summary of outcomes from analyses of the All-Japan out-of-hospital cardiac arrest registry

Author	Hagihara	Nakahara	Nakahara
Period	2005–2008	2007–2010	2007–2010
Subset	NA	Shockable	Non-shockable
Total number of cases	417,188	14,943	81,136
ROSC			
ROSC with adrenaline (unadjusted)	18.5%	21.6%	18.5%
ROSC without adrenaline (unadjusted)	5.7%	28.1%	5.7%
Adjusted OR (95% CI)	3.75 (3.59–3.91)^a^	NA	NA
One-month survival			
One-month survival with adrenaline (unadjusted)	5.4%	16.5%	3.9%
One-month survival without adrenaline (unadjusted)	4.7%	28.8%	4.2%
Adjusted OR (95% CI)	0.54 (0.43–0.68)^a^	1.34 (1.12–1.60)^b^	1.72 (1.45–2.04)^b^
CPC 1–2			
CPC 1–2 with adrenaline (unadjusted)	1.4%	6.9%	0.6%
CPC 1–2 without adrenaline (unadjusted)	2.2%	19.8%	1.5%
Adjusted OR (95% CI)	0.21 (0.10–0.44)^a^	1.01 (0.78–1.30)^b^	1.57 (1.04–2.37)^b^

^a^Data adjusted for propensity and all covariates

^b^Time-dependent propensity score-matched data

Another analysis of the same Japanese nationwide OHCA registry, but using a different period (2007 and 2010), showed that among patients receiving adrenaline, the unadjusted rate of ROSC was higher in those with an initial non-shockable rhythm (18.5 vs 5.7%) but lower in those with an initial shockable rhythm (21.6 vs 28.1%) [16] (Table 1). The unadjusted survival rates (at one month or to discharge) and rates of survival CPC 1–2 in all patients were lower in those receiving adrenaline. The authors identified 1990 propensity-matched pairs of patients with and without adrenaline with an initial shockable rhythm, and 9058 propensity-matched pairs of patients with an initial non-shockable rhythm. In contrast to the Hagihara study [15], after propensity matching, the aOR for survival favoured adrenaline for both shockable (aOR 1.36, 95% CI 1.13–1.63) and non-shockable rhythms (aOR 1.78, CI 1.49–2.13). The aORs for survival with CPC 1–2 in those with non-shockable rhythms (OR 1.55, CI 0.99–2.41) and those with shockable rhythms (aOR 1.02, CI 0.78–1.33) indicate no significant difference with and without adrenaline. Nakahara and colleagues [16] used a time-dependent propensity analysis which may account for the contradictory findings between their study and that of Hagihara and co-investigators. Time-dependent propensity analysis better adjusts for what has recently been described as ‘resuscitation time bias’ where interventions such as injection of adrenaline are more likely to be implemented the longer the duration of cardiac arrest, and longer durations of cardiac arrest are associated with worse outcome [17].

A third analysis of the Japanese nationwide registry, this time covering the period 2009–2010, identified 209,577 OHCA [18]. Among the 15,492 patients who had an initial shockable rhythm, the rate of ROSC, one-month survival and one-month CPC 1–2 was 27.7, 27.0, and 18.6% in those who did not receive adrenaline and 22.8, 15.4, and 7.0% in those who did receive adrenaline (all *p* < 0.001). In the 194,085 patients who initially had a non-shockable rhythm, the rate of ROSC and one-month survival was 3.0%, and 2.2% in those who did not receive adrenaline, and 18.7 and 3.9% in those who did receive adrenaline (both, *p* < 0.001). There was no significant difference in one-month CPC 1–2 between the two groups. Injection of adrenaline within 20 min of onset of CPR was associated with better survival. For non-shockable rhythms, injection of adrenaline within 10 min and 10–19 min of the onset of CPR was associated with increased one-month survival (aOR 1.78, 95% CI 1.50–2.10 and aOR 1.29, CI 1.17–1.43, respectively). Delayed injection of adrenaline was associated with worse neurological outcomes at one month (aOR 0.63, 95% CI 0.48–0.80 and aOR 0.49, CI 0.32–0.71) for adrenaline injected at 10–19 min and greater than 19 min, respectively. Several studies have shown that early adrenaline administration is associated with better outcomes compared with later adrenaline (see ‘Adrenaline timing’ below).

A Paris study, including all patients with OHCA who achieved ROSC and were admitted to a single centre between 2000 and 2012, found that 17% of patients who received adrenaline had a favourable neurological outcome (CPC 1–2) while 63% of patients who did not receive adrenaline had a CPC 1–2 [19]. After adjusting for known confounders, use of adrenaline was associated with a worse neurological outcome (aOR for favourable neurological outcome 0.32, 95% CI 0.22 to 0.47), even after adjusting for in-hospital interventions. Although the authors made considerable effort to adjust for confounders, the observational nature of this study precludes any firm conclusion on causality.

The effect of adrenaline can be inferred from a before-after trial in Ontario, Canada, which studied the impact of introducing prehospital advanced life support (ALS) to an optimised basic life support automated external defibrillation (BLS-AED) system [20]. The ALS phase included tracheal intubation and intravenous drugs. Of the 4247 patients enrolled in the ALS phase, 95.8% received adrenaline. Patients in the ALS phase had higher rates of ROSC (18.0 vs 12.9%, *p* < 0.001) and survival to hospital admission (14.6 vs 10.9%, *p* < 0.001) but no difference in survival to hospital discharge (5.1 vs 5.0%, *p* = 0.83) [20]. The limitation of this study is that it is difficult to separate the impact on outcome of tracheal intubation and injection of adrenaline. For example, any beneficial effect of adrenaline could be offset by harm caused by tracheal intubation, and vice versa. Determining the impact of single interventions when they are delivered as components of a package of care is challenging.

**Fig. 1:**
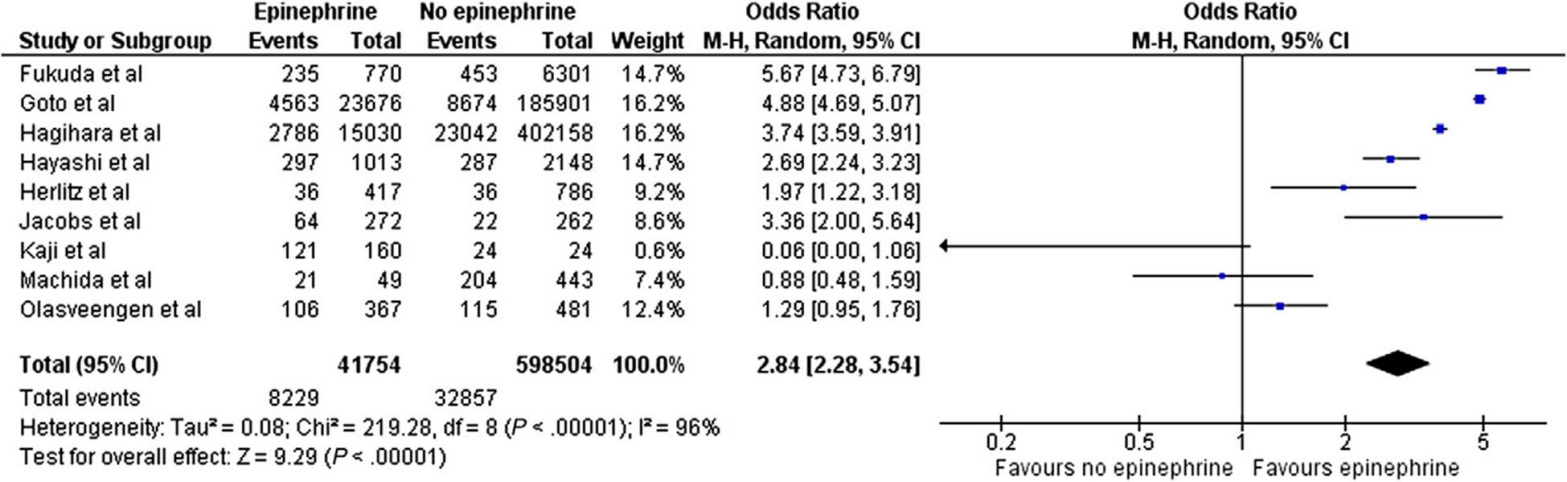
Forrest plot comparing ROSC for those who did, and did not, receive adrenaline (epinephrine)

**Fig. 2:**
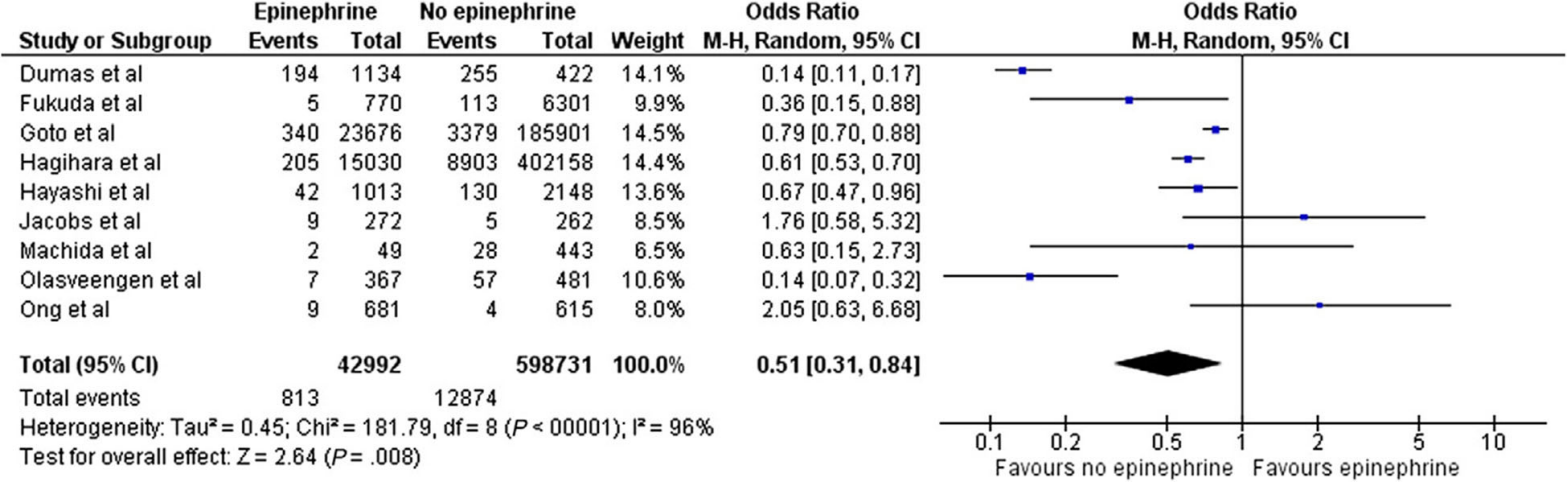
Forrest plot comparing favourable neurological outcome (CPC 1–2) for those who did, and did not, receive adrenaline (epinephrine)

A recent systematic review and meta-analysis including 13 observational studies and one randomised controlled trial, with 655,653 OHCA patients, found that the administration of adrenaline before hospital arrival was associated with an increase in ROSC (OR 2.84, 95% CI 2.28–3.54, *p* < 0.001), but was also associated with an increase in the risk of poor neurological outcome at hospital discharge (OR 0.51, 95% CI 0.31–0.84, *p* < 0.01), without affecting survival at one month (Figs. 1 and 2) [21].

In summary, these observational data suggest that adrenaline increases the rate of ROSC, but may have detrimental effects on overall survival, particularly neurologically intact survival. It appears to have greatest benefit—or least harm—in patients with cardiac arrest with an initial non-shockable rhythm.

### Adrenaline timing

An analysis of 25,095 adult in-hospital cardiac arrest (IHCAs) with an initial non-shockable rhythm in the American Heart Association Get with the Guidelines-Resuscitation (AHA GWTG-R) registry between 2000 and 2009 identified an association between survival and time to injection of adrenaline [22]. Time to adrenaline administration was analysed by 3-minute intervals, with an aOR of survival to hospital discharge of 1.0 for 1–3 min as the reference group. The aOR for survival to hospital discharge was 0.91 (95% CI 0.82–1.00, *p* = 0.055) for 4–6 min, 0.74 (95% CI 0.63–0.88, *p* < 0.001) for 7–9 min, and 0.63 (95% CI 0.52–0.76, *p* < 0.001) for over 9 min. The results were similar for good neurological survival.

Another analysis of the AHA GWTG-R registry included patients with an initial shockable rhythm who were defibrillated within 2 minutes of the cardiac arrest and who remained in a shockable rhythm after defibrillation [23]. The authors focused on the patients who were given adrenaline within 2 minutes after the first defibrillation, which is counter to the guidelines of the AHA and European Resuscitation (these organisations recommend adrenaline delivery only after the second or third shocks, respectively). Of 2978 propensity-matched patients, 1510 received adrenaline within 2 minutes of defibrillation and this intervention was associated with decreased odds of survival (OR 0.70, 95% CI 0.59–0.82, *p* < 0.001). Early injection of adrenaline was also associated with a decreased rate of ROSC (OR 0.71, 95% CI 0.60–0.83, *p* < 0.001) and good functional outcome (OR 0.69, 95% CI 0.58–0.83, *p* < 0.001). As well as the potential decrease in cerebral and coronary microcirculatory flow, it is possible that the increase in myocardial oxygen demand associated with adrenaline may be particularly harmful in the first few minutes of a VF cardiac arrest.

A further analysis of the AHA GWTG-R registry evaluated the impact on outcome of time to adrenaline administration among children (age < 18 years) with IHCA and an initial non-shockable rhythm [24]. Among 1558 children, 31.3% survived to hospital discharge. Although the median time to first adrenaline dose was 1 minute (interquartile range 0–4), multivariate analysis identified that longer time to adrenaline administration was associated with lower risk of survival to discharge, with a risk ratio (RR) of 0.95 per minute delay (95% CI 0.93–0.99), as well as a lower risk of survival with favourable neurological outcome, RR 0.95 per minute delay (95% CI 0.91–0.99). Children in whom the time to adrenaline administration was longer than 5 minutes had a lower risk of survival to discharge compared with those given adrenaline within 5 minutes (21.0 vs 33.1%, aRR 0.75, 95% CI 0.60–0.93, *p* = 0.01).

Another analysis of the Japanese nationwide registry between 2008 and 2012 included 119,639 patients with a witnessed OHCA [25]. The 20,420 patients who received adrenaline were divided into four groups based on timing of adrenaline administration: early adrenaline (5–18 min), intermediate adrenaline (19–23 min), late adrenaline (24–29 min), and very late adrenaline (30–62 min). Multiple logistic regression analyses and aORs were determined for CPC 1–2 at one month, and for ROSC. Overall, the adrenaline group had a higher rate of ROSC (18 vs 9.4%) but a lower rate of CPC 1–2 (2.9 vs 5.2%). In comparison with the late group, CPC 1–2 was highest in the early adrenaline group (aOR 2.49, 95% CI 1.90–3.27), followed by the intermediate group (aOR 1.53, 95% CI 1.14–2.05); the very late adrenaline group had the worst neurological outcomes (in comparison with the late group: aOR 0.71, 95% CI 0.47–1.08).

Other observational studies have shown that adrenaline is rarely given very early in a cardiac arrest. In a literature review where drug delivery time was reported in 7617 patients, the mean time to first drug delivery by any route was 17.7 min [26]. Another US retrospective study of 686 patients reported similar findings—the mean time to adrenaline administration was 14.3 min, while those who received early adrenaline (within 10 min) were more likely to have ROSC (32.9 vs 23.4%, OR 1.59, 95% CI 1.07–2.38), although there was no significant difference in survival to discharge [27].

In summary, these observational data indicate that earlier use of adrenaline is associated with better outcomes than later use of adrenaline, but in patients with an initial shockable rhythm, administration of adrenaline within 2 minutes of the first defibrillatory shock may be detrimental.

### Adrenaline dose

**Fig. 3:**
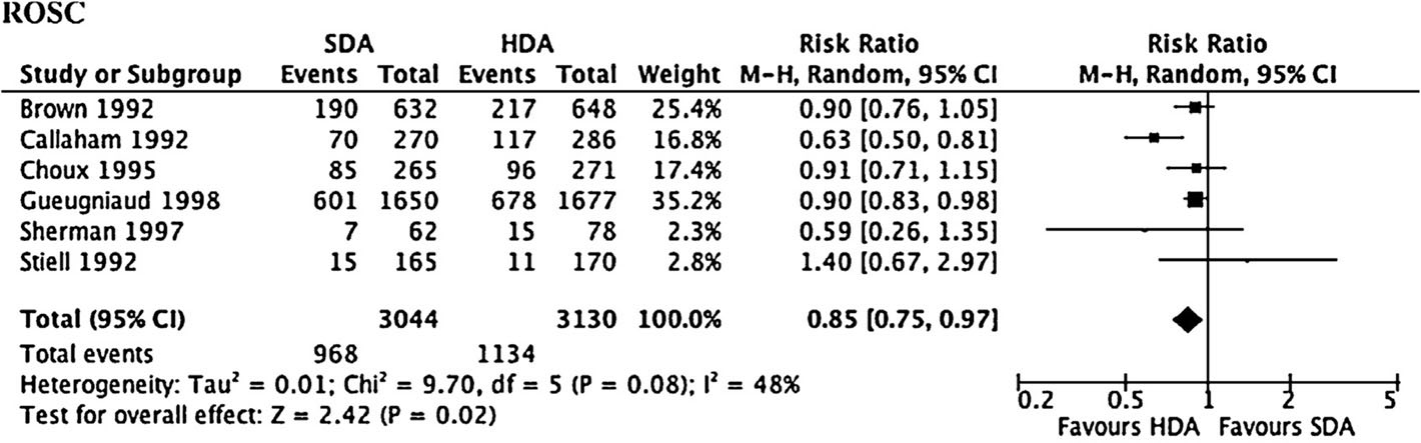
Forrest plot comparing ROSC for those who received high-dose adrenaline (*HDA*) compared with standard dose adrenaline (*SDA*)

**Fig. 4:**
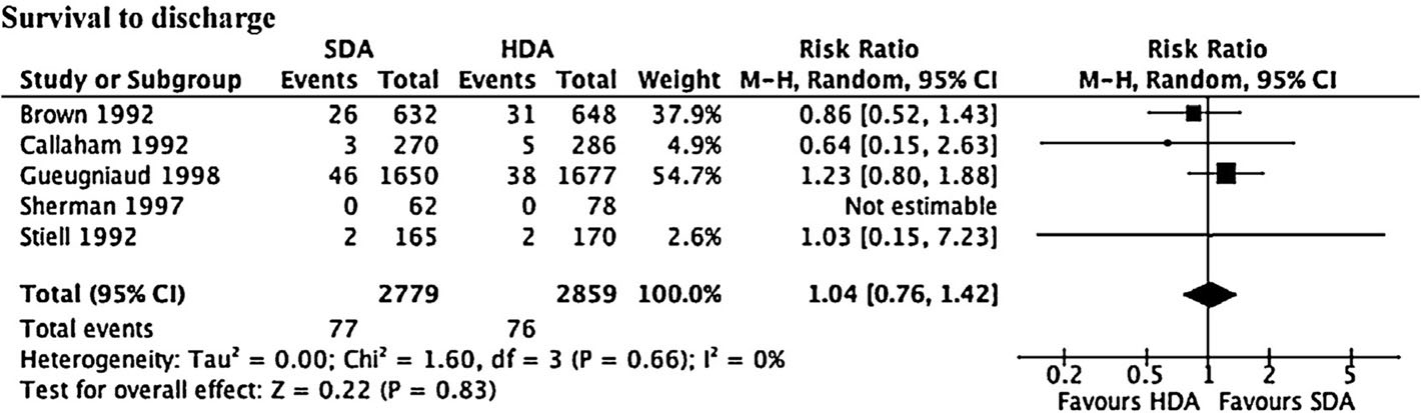
Forrest plot comparing survival to hospital discharge for those who received high-dose adrenaline (*HDA*) compared with standard dose adrenaline (*SDA*)

The optimal dose of adrenaline remains unknown. A meta-analysis of six randomised controlled trials (RCTs) comparing standard dose adrenaline (1 mg; SDA) with high-dose adrenaline (>  1 mg; HDA) found that SDA had a lower rate of ROSC (RR 0.85, 95% CI 0.75–0.97, *p* = 0.02) (Fig. 3), and survival to admission (RR 0.87, 95% CI 0.76–1.00, *p* = 0.049). However, there was no difference in survival to discharge (RR 1.04, 95% CI 0.76–1.42; Fig. 4) or neurologically favourable survival (RR 1.20, 95% CI 0.74–1.96) [28].

A recent before–after study of 2255 patients with non-traumatic OHCA compared different doses of adrenaline. Initially, 1 mg adrenaline was given at 4 min, followed by additional 1 mg doses every 2 min in those with non-shockable rhythms, and every 8 min in those with shockable rhythms [29]. During the intervention period, 0.5 mg of adrenaline was given at 4 and 8 min, followed by every 2 min in those with non-shockable rhythms, and every 8 min in those with shockable rhythms. Although the dose of adrenaline per patient reduced during the intervention period, there was no difference in survival to hospital discharge or favourable neurological outcome in either the shockable or non-shockable groups.

## Adrenaline dosing intervals

A review of 20,909 IHCAs from the AHA GWTG-R defined the adrenaline average dosing interval as the time between the first adrenaline dose and the resuscitation endpoint, divided by the total number of adrenaline doses received after the first dose [30]. Compared with an average dosing interval of 4 to < 5 min per dose, survival to hospital discharge was higher in patients with longer dosing intervals: aOR 1.41 (95% CI 1.12–1.78) for 6 to < 7 min/dose; aOR 1.30 (95% CI 1.02–1.65) for 7 to < 8 min/dose; aOR 1.79 (95% CI 1.38–2.32) for 8 to < 9 min/dose; aOR 2.17 (95% CI 1.62–2.92) for 9 to < 10 min/dose. A much smaller single-centre study of 896 IHCAs in Taiwan also found an association between shorter adrenaline dosing intervals and worse outcome [31].

An analysis of 1630 IHCAs among children in the same registry categorised average dosing intervals as 1–5 min, > 5 to < 8 min, and 8 to < 10 min/dose [32]. Compared with a reference of 1–5 min/dose, the aOR for survival to hospital discharge was 1.81 (95% CI 1.26–2.59) for > 5 to < 8 min/dose, and 2.64 (95% CI 1.53–4.55) for 8 to < 10 min/dose.

In summary, although high-dose adrenaline had no apparent benefit over standard-dose adrenaline, a higher rate of survival to hospital discharge was associated with longer adrenaline dosing intervals.

## Clinical randomised controlled trials

In a study from Norway, 851 OHCA patients were randomised to receive either ALS with IV access and drugs as indicated (IV group) or ALS with IV access delayed until 5 min after ROSC (no IV group) [33]. Eighty percent of the patients in the IV group received adrenaline during resuscitation. In the 286 patients whose initial rhythm was shockable (VF/pVT), there were no differences between the groups in the rates of ROSC, survival to ITU admission, or survival to hospital discharge. In the 565 patients with an initial non-shockable rhythm (asystole or pulseless electrical activity (PEA)), those in the IV group had higher rates of ROSC (29 vs 11%, *p* < 0.001) and survival to ITU admission (19 vs 10%, *p* = 0.003), but survival to hospital discharge was similar (2 vs 3%, *p* = 0.65).

A post hoc analysis of this study compared outcomes for patients actually receiving adrenaline with those not receiving adrenaline [34]. Patients receiving adrenaline had a higher rate of hospital admission (OR 2.5, 95% CI 1.9–3.4) but lower rate of survival to hospital discharge (OR 0.5, 95% CI 0.3–0.8) and lower rate of neurologically intact survival (OR 0.4, 95% CI 0.2–0.7).

A double-blind placebo-controlled RCT from Western Australia randomised 534 patients to ALS with and without adrenaline. The adrenaline group had a higher rate of hospital admission (25.4 vs 13.0%, OR 2.3, 95% CI 1.4–3.6) but survival to hospital discharge was not statistically different between the groups (4 vs 1.9%, *p* = 0.15). The effect of adrenaline on pre-hospital ROSC was particularly marked in non-shockable rhythms (OR 6.9, 95% CI 2.6–18.4) than in shockable rhythms (OR 2.4, 95% CI 1.2–4.5). With the exception of two patients in the adrenaline group, all survivors had good neurological outcomes (CPC 1–2) [35].

In summary, these data from prospective clinical trials suggest that adrenaline increases the rate of ROSC, but not long-term survival or neurologically favourable survival.

## Ongoing studies

The PARAMEDIC-2 trial (Pre-hospital Assessment of the Role of Adrenaline: Measuring the Effectiveness of Drug administration In Cardiac arrest) has recently finished recruiting more than 8000 patients. This individually randomised, double-blind, placebo-controlled trial included OHCA patients in whom ALS was initiated, while excluding patients in cardiac arrest from anaphylaxis or life-threatening asthma, under-16 year olds, and those who were pregnant. Adrenaline and placebo were prepared in identical syringes and placed in pre-randomised packs of ten syringes. Outcomes will be survival to 30 days, hospital discharge, 3, 6, and 12 months, health-related quality of life, and neurological outcomes at hospital discharge and 3 and 6 months [36]. The results of this study will be reported in 2018.

## Conclusions

Although the administration of adrenaline remains one of the most common ALS interventions, and likely increases rate of ROSC after cardiac arrest, its effect on long-term outcomes is far less certain. Several animal studies indicate that whilst global blood flow to vital organs is generally increased, microcirculatory flow may be made worse by adrenaline. Many clinical observational studies document an association between the injection of adrenaline and worse long-term outcomes, yet others show an association between early injection of adrenaline and better long-term outcome. Ultimately, it is hoped that the recently completed large RCT comparing adrenaline with placebo will provide some clarity on the role of adrenaline, if any, in the treatment of cardiac arrest.
